# What Do Older Adults with Frailty and Their Caregivers Want from Advance Care Planning Discussions? A Descriptive Qualitative Study

**DOI:** 10.3390/healthcare14010002

**Published:** 2025-12-19

**Authors:** Robin Urquhart, Cynthia Kendell, Jessica Vickery, Elias Hirsch

**Affiliations:** 1 Department of Community Health and Epidemiology, Dalhousie University, Halifax, NS B3H 1V7, Canada; jvickery@dal.ca; 2 Department of Surgery, Nova Scotia Health Authority, Halifax, NS B3H 2Y9, Canada; eliashirsch11@gmail.com; 3 Department of Medicine, Dalhousie University, Halifax, NS B3H 2Y9, Canada; cynthia.kendell@nshealth.ca

**Keywords:** advance care planning, frailty, caregivers, quality of life, decision-making, autonomy

## Abstract

**Introduction:** Advance care planning (ACP) may facilitate person-centered, goal-concordant care for this population. However, we know little about what older adults expect from ACP discussions (that is, the outcomes they expect or desire). We sought to explore patient and family views on the outcomes they consider most pertinent to ACP discussions for older adults living with frailty. **Methods:** This qualitative descriptive study used semi-structured telephone/video-conferencing interviews with older persons with frailty (65+ years) and their family/friend caregivers from across Canada. All interviews took place in person or via telephone or Zoom with healthcare professionals, depending on the participant’s preference, and were conducted by a researcher with experience in qualitative methods. Data analysis involved coding, grouping, detailing, and comparing the data, using techniques commonly employed in descriptive qualitative research. **Results:** Nine participants took part in this study: two were individuals living with frailty, and seven were caregivers of persons with frailty. They described three desired outcomes of ACP: (1) maximizing comfort and quality-of-life; (2) enhancing confidence in decision-making, which would heighten families’ level of reassurance as their loved one’s function and health status declines; and (3) maintaining autonomy so that people can live and die on their own terms. **Conclusions:** Ultimately, research and clinical care should tailor their ACP initiatives and evaluation metrics to align with what patients and families deem most important. The outcomes described by participants of this study should be further investigated and refined in future research.

## 1. Introduction

From a health system and societal perspective, the increasing prevalence of frailty may be the most concerning manifestation of population aging [1]. Frailty is highly prevalent among older, community-dwelling adults worldwide: a recent pooled analysis of 46 observational studies from 28 countries suggests that 1 in 6 community-dwelling older adults are frail [2]. In Canada, 24% of community-dwelling adults age 65 years and older are frail, with another 32% at high risk of becoming frail [3]. Frailty is associated with greater use of health care services [4] and is the leading pathway to death (24%) amongst older adults, more common than organ failure (21%), cancer (19%), or dementia (14%) [5,6].

Persons with frailty deserve person-centered, goal-concordant care as they near the end of life. Research demonstrates this is not the reality for many Canadians. For example, as older adults with frailty experience considerable loss of capacity in later life, most report their desire for treatment aimed at maximizing quality of life rather than prolonging life [7,8,9]. However, the use of invasive life-sustaining technologies amongst older Canadians with frailty is increasing [10,11], and older adults account for more than 50% of patients admitted to intensive care units in Canada [12].

Advance care planning (ACP) may facilitate person-centered, goal-concordant care for this population. As defined in an international Delphi consensus process, ACP “is a process that supports adults at any age or stage of health in understanding and sharing their personal values, life goals, and preferences regarding future medical care” [13]. The goal of ACP is to prepare people, and their substitute decision-maker(s), for future in-the-moment treatment decisions so that people receive medical care consistent with their values and preferences during serious illness. While studies have shown that ACP results in improved patient, family, and system outcomes, systematic reviews of ACP have demonstrated inconsistent findings [14]. One of the methodological challenges in this field concerns the various outcomes that have been used to evaluate the effectiveness of ACP programs, with little consistency in how these outcomes are measured [15,16,17]. Another limitation of research to date is that, apart from satisfaction with communication or medical care received, few researchers have evaluated ACP from a person (patient or family) perspective [18].

To optimize ACP research for older adults with frailty, there is a need to understand what they and their family/friend caregivers expect from the ACP process. This will permit the selection of appropriate and relevant outcomes for ACP research. Understanding the outcomes important to patients/families is also key to tailoring interventions and designing trials in ways that address the things that matter most to patients and their families. Numerous studies have found that the outcomes important to patients are overlooked or viewed unimportant by research teams [19,20,21,22]. The objective of this study was to explore patient- and family-relevant outcomes pertinent to ACP discussions for those living with frailty.

## 2. Materials and Methods

### 2.1. Study Design and Reporting Standards

We conducted a qualitative descriptive study [23,24], using semi-structured telephone interviews with older persons with frailty and their family/friend caregivers from across Canada. Qualitative description summarizes and describes the informational content of the data with minimal, albeit some, interpretation. We selected this approach given the exploratory nature of this study, wherein we hoped to provide a straightforward description of people’s experiences and expectations of ACP, using their own language and with low inference. As such, we also did not use an a priori theoretical framework to situate the study. Study approval was granted by the Nova Scotia Health Research Ethics Board. The Consolidated Criteria for Reporting Qualitative Research was used and is presented in File S1.

### 2.2. Setting and Participants

Eligibility criteria for participation were broad. Specifically, study participants must have been community-dwelling older adults (65 years of age or older) with frailty (operationalized as someone experiencing a loss of energy, physical ability/function, and/or cognition) or family/friend caregivers of older adults with frailty (operationalized in the same way). Participants were recruited by distributing study information through patient and caregiver organizations and networks across Canada. Interested persons were instructed to contact a research coordinator by telephone or email to acquire further information about the study. No attempt was made to verify a person’s self-reported frailty status given we did use clinic-based methods to recruit and did not have access to participants medical records or charts. When the person opted to participate, the coordinator arranged a time to conduct the subsequent interview. Written informed consent was obtained and confirmed by the coordinator prior to interview commencement. The recruitment period was open between 28 January 2021, and 23 August 2023.

### 2.3. Data Collection

Prior to participation, participants received a brief summary to describe ACP to ensure people understood the topic for discussion. This description was also used during the interview to confirm and/or clarify their understanding. All interviews took place in person or via telephone or Zoom for Healthcare, depending on the participant’s preference. A researcher [CK, EH, or JV] with prior experience in qualitative methods conducted all interviews. A senior researcher [RU] reviewed all transcripts after each interview and debriefed with the interviewer if any deviations in interviewing were noted. There was no relationship between the researchers and any participants prior to study commencement. The interview guide (see File S2) was developed based on the study objective using practical guidance from Patton [25] and Rubin and Rubin [26]. Questions were not pilot tested in advance, but the interview guide was adapted over the course of the first two interviews, aligned with qualitative methods. Participants were asked to reflect on what ACP would ideally *do* for persons with frailty and their family/friend caregivers. Each interview was audiotaped to ensure the data were captured and retrievable in true form, and transcribed verbatim by an experienced transcriptionist with >20 years of transcription experience. The audiotapes and transcripts were supplemented with field notes, allowing the researcher to highlight particularly insightful data and to capture personal reflections. No repeat interviews were conducted.

### 2.4. Data Analysis

Data analysis involved coding, grouping, detailing, and comparing the data, using techniques commonly employed in descriptive qualitative research [23,24]. To begin this work, two researchers [JV, RU] independently reviewed and inductively coded two transcripts to identify salient concepts. In doing so, they developed a coding framework to guide ongoing analyses. One researcher [JV] used this framework to code the remaining transcripts, with another researcher [RU] reviewing and questioning all coding. A third researcher [CK] reviewed selected coded transcripts as a check on coding, including consistency with applying codes to emerging concepts. Meetings between JV, RU, and CK were held as needed to discuss coding. Salient codes were collapsed into larger categories to identify overarching themes (i.e., desired outcomes). The analysis was performed manually, with the assistance of qualitative software (NVivo version 13) for data management and to facilitate comparison and synthesis of codes. An audit trail was maintained, starting with coding of the first transcript to final analysis. Inter-coder reliability was not assessed; rather discussions with the full research team occurred to facilitate questioning of the data and its analyses, and to come to agreement on the final themes. Peer debriefing of emerging and final themes was carried out with colleagues also conducting research on ACP for persons living with frailty.

### 2.5. Reflexivity and Researcher Characteristics

Two researchers [RU, CK] had prior research experience in ACP, both in the cancer and frailty contexts; this includes previously developing and implementing ACP training for healthcare providers [RU]. This prior experience may have influenced research conduct (e.g., interview questions) as well as our interpretation and framing of the findings. While we believe our “closeness” to ACP was a strength, helping us probe in a more in-depth way and view the data in a more nuanced way, it may have also led us to focus on what we felt was important and therefore how we framed the final themes. To mitigate these potential biases, we documented our coding process and related decisions (audit trail), met to question our coding and emerging categories/themes, and undertook peer debriefing with colleagues outside of the research team.

## 3. Results

Nine individuals participated in the study: two were individuals living with frailty (both women) and seven were caregivers of persons with frailty (five women, two men). Caregivers were all close family members (spouses or children). All participants were community-dwelling individuals, living at home. Three participants reported not having an ACP discussion prior to the interview; for the remaining participants, discussions involved family members and not formal discussions with their healthcare teams.

Participants described three desired outcomes of ACP: (1) maximizing quality of life, (2) confidence in decision-making, and (3) maintaining autonomy.

### 3.1. Maximizing Quality of Life

Most participants discussed the desire to maximize comfort and enjoyment as their (or their loved one’s) function and overall health decline, and believed that ACP is a tool to help people achieve this. By elucidating a plan ahead of time, persons with frailty and their families can focus on maximizing one’s quality of life rather than worrying about medical care. As one participant succinctly said, “*Advance care planning is him having his favorite things*” [P5]. This participant went on to describe the following decision, based on their loved one’s wishes:

“We’ve chosen not to treat it except for Zometa, a bone building drug. Because the steroids and the treatment for the myeloma would bring on the dementia faster, and as we say sort of tongue in cheek, ‘What are we saving him for? The joys of Lewy body?’”

[P5]

Other participants also referred to pain and symptom management as they discussed the desire to maximize comfort. As one participant described:

“I think it gives us clear direction on moving forward, and it kind of has the plan in place so that we know what next steps are to a certain degree. I mean, you never really know, but, and you’re never truly prepared, but to focus on the good life and that they’re not in pain anymore, I think that’s the biggest thing.”

[P4]

### 3.2. Confidence in Decision-Making

Participants discussed three key issues with respect their expectation that ACP would enhance confidence in decision-making when decisions have to be made. First, most participants discussed the importance of knowing their options when it comes to multiple healthcare, practical, and legal decisions, and how this allows the patient (and/or family) to make the best decisions possible given the circumstances. Participants acknowledged that such decisions are often unique to one’s individual and family circumstances but also that discussions are key to understanding these differences and allowing one to prepare before a crisis occurs. One participant described it in this way:

“Doctors are reluctant to tell you the range of things that it could look like in the future. Everybody’s different. Well, they are, but there’s a range. What’s this range? This range. And I’ll figure out where it goes. So, people that I rely on to tell me what they’ve seen, what the options are so I can prepare. So advance care planning is me anticipating what’s going to happen anyway. This is how I look at it.”

[P5]

Second, all caregiver participants discussed how having an advance care plan in place would heighten their level of reassurance as their loved one’s function and health status declined. As two participants said:

“[Planning] gives me comfort that I can handle it. I can handle the next thing until I get there and then figure that out from there. I can’t imagine walking through this without thinking about the future. That’s crazy.”

[P5]

“I think it would be very stressful on everyone if there wasn’t a plan. You have to go through all the scenarios that you have to go through.”

[P8]

Participants discussed how an advance care plan would provide confidence that they are making decisions that are compatible with their loved one’s wishes, and would reduce uncertainty and conflict during episodes of care. In this way, one participant described advance care planning as a gift that helps loved ones during times of distress:

“I approach advance care planning as a gift. It’s a gift you give yourself because you’ll hopefully get the treatment that you want, and the treatments you don’t want are not given to you. And it’s also a gift you give to your family because it gives them kind of a peace knowing what you want and not. It helps the family members, who sometimes in a family meeting in the ICU or something that they can be quite intimidating. … And I also would say that even when somebody has died, I’ve seen that gift continue after the death because folks have showed up for the reading of the will and then they challenge the substitute decision maker. ‘Why didn’t you have more aggressive care? Why didn’t you have CPR? Why didn’t you have a feeding tube?’ And I’ve seen people pull out the advance care plan and say, ‘I did what my spouse wanted.’”

[P2]

Some participants also discussed how they have realized the value of such discussions due to past experiences of conflict:

“We talk about that stuff because I had a horrible experience with my brothers when my parents died, so I don’t want them going through that.”

[P6]

Third, participants discussed how ACP helps people know how to respond when crises happen because they have had prior discussions about values and preferences–even if the crisis differs from what was discussed. In such decisions, participants discussed how prior conversations and planning had the potential to mitigate panicked decisions or decisions first contemplated and made during times of acute mental stress. As two participants described:

“A goal, too, I think is to try to as much as possible to not have to make decisions in a crisis. If you can think of these things before the crisis, ask questions before the crisis. Hopefully when the crisis comes, then you can make decisions you won’t regret.”

[P2]

“[Caregiving is] definitely stressful on you. I think it would be nothing compared to the stress you would have if you didn’t have things planned out as you do. How can you make decisions because when somebody falls ill, your mental health isn’t the same? So, your ability to make decisions wouldn’t be as effective as if they were made beforehand when your mental health was better.”

[P4]

Other participants discussed specific examples of how prior discussions helped them in specific health care decision-making. One participant, whose spouse was admitted to the emergency department with sepsis, said:

“So when the intensivist asked me a year ago, “I have something sensitive to talk about,” because [my wife and I] had the conversation, it was easier. There was no fear. There’s not fear in terms of am I doing something that’s against her wishes?”

[P2]

### 3.3. Maintaining Autonomy

Whether discussing medical care (e.g., do not resuscitate orders) or practical concerns (e.g., finances, housing arrangements), participants continually discussed the importance of making upstream decisions that allowed persons to maintain autonomy and live and die on their own terms. As one participant said, “*With this [disease], it can get really debilitating, and I don’t want to be that. That’s not who I am*” [P6]. Participants emphasized that the need for autonomy continued even when someone had cognitive decline. As a caregiver participant of a loved one with dementia said, “*I feel at the front end, he needs to be treated like an adult who is fully in charge of his life. So, I ask his permission for things and he’s very comfortable with all of that*.” [P5]

## 4. Discussion

This study sought to understand patient- and family-relevant outcomes pertinent to ACP discussions for those living with frailty. Such knowledge is imperative to selecting meaningful and salient outcomes for future research and to tailoring ACP programs and interventions to address the things that matter most to patients and their families. When asked what they expected of and desired from ACP, participants elucidated outcomes related to maximizing one’s quality of life as health declines, enhancing confidence in decision-making, including in crisis events, and maintaining autonomy so that a person can live and die on their terms. These patient- and family-identified outcomes represent a starting point for researchers to expand upon in future research and to select measures that assess ACP from a patient- and family-perspective.

Although the majority of older adults with frailty report they would welcome ACP discussions, the literature suggests that most have not had this opportunity [27]. Similarly, research related to frailty and ACP has lagged behind our understanding of ACP in relation to other conditions (e.g., cancer). Despite this, research has shown that maintaining wellness and living well in the moment are important goals for older adults with frailty in the context of ACP and planning for end-of-life care [28,29]. Interviews of bereaved relatives who were involved in a cluster randomized controlled trial of ACP in older adults with frailty revealed that most (90%) of those in the intervention group appreciated ACP and felt appropriately prepared for decision-making [30]. These goals and described outcomes align well with findings from this study.

From a research perspective, the inconsistency in ACP outcomes–both the outcomes used and how they are measured–has been recognized by others [31,32]. In fact, the heterogeneity in outcomes led Sudore et al. to conduct an international Delphi study of clinical, academic, and policy experts to generate consensus-based outcomes to evaluate ACP programs and interventions [31]. The resulting ACP Outcomes Framework includes process, action, quality of care, and health care outcomes, and should act as a guiding framework toward standardizing outcomes in this field. The five top-rated outcomes in the Delphi study were as follows: care consistent with goals, surrogate designation, surrogate documentation, discussions with surrogates, and documents with recorded wishes are accessible when needed. While many of these action-oriented outcomes may lead to the outcomes delineated in this current study, they do not necessarily reflect the patient or family experience as they anticipate or go through the ACP process. Indeed, outcomes related to control over decision-making (autonomy) and quality-of-life received a much lower overall ranking (e.g., decision control preferences = overall ranking of 51/75; peace = overall ranking of 50/75; self-rated quality of life = overall ranking of 53/75). Future work should seek to incorporate patient and family views on the most salient and meaningful outcomes to evaluate ACP programs. The results of the current study provide a foundation to begin such investigation.

A recent scoping review used the ACP Outcomes Framework [31] to understand the range of interventions and outcomes employed by researchers since 2010 [32]. The authors found 170 outcomes evaluated across the 69 studies included in the review, demonstrating the high heterogeneity in outcomes measurement. The authors also found that process- and action-oriented outcomes (e.g., documentation) were often positive, yet outcomes related to quality of care and health outcomes (e.g., goal concordance and quality of life) were often mixed. For example, while 100% of the outcomes assessing satisfaction with communication were positive, none of the quality-of-life outcomes were positive. The latter finding may reflect the complexity of quality-of-life measurement or that current instruments are not well suited to those with declining function and who are nearing the end of life. Outcomes that are arguably less complex and perhaps more proximal to individuals’ everyday lives (e.g., enjoying daily activities, living without pain, reassurance with decision-making) may be more meaningful and actionable for patients and families as their health declines. Alternatively, while people may expect ACP to improve quality of life, adequate health system resources and capacity (e.g., ability to provide medical equipment or care at home) are also necessary to meet this goal.

Other than quality-of-life and symptom measures, there are few existing patient/family reported outcome and experience measures related to ACP. Researchers evaluating ACP have assessed patient/family satisfaction, quality of communication, patient decisional conflict, and the family’s confidence in end-of-life decision-making using validated tools [33,34,35,36]. However, tools are typically not specific to ACP conversations/processes. In a recent systematic review of patient-reported outcome measures for advance care planning in older adults [37], the authors identified 202 patient-reported outcome measures from 74 ACP intervention studies; these were wide ranging in scope and included measures related to quality of communication, decisional conflict, and perceptions around autonomy. After excluding outcomes related specifically to health status (e.g., quality of life and mental status) and removing duplications, the authors appraised the psychometric properties of 86 measures. None of the ACP-specific patient reported outcome measures fully met the Consensus-based standards for the selection of health measurement instruments (COSMIN) for measurement development. Moreover, only 15 of these measures were used more than once across studies (n = 2–14). The findings of this review suggest that more work is needed to develop validated patient/family reported outcome measures for future ACP evaluation and research.

This study has a number of limitations. The first is the low number of participants. While this study was exploratory in nature, our final sample size was 9, which is relatively low. While the dataset was rich, with participants articulating their desires and expectations related to ACP, it is difficult to determine whether saturation was fully achieved. Moreover, due to the small sample size, we analyzed the dataset in its entirety rather than separating the data into older adults with frailty and caregiver subsets. Still, prior researchers have found that saturation can be achieved with nine to twelve participants [38,39], and that the basic features of meta themes are often present with as few as six interviews [39]. Second, only two of the participants identified as men and, given the gendered nature of caregiving, it is possible that there are differences between men’s and women’s expectations that could not discerned in this study. Related, the importance of specific outcomes may differ across cultural contexts, which may limit the transferability of findings. Third, data collection occurred during the global COVID-19 pandemic, including during the emergence and predominance of the Omicron strain. We found recruitment during this period particularly challenging, possibly due to pandemic restrictions, burden, and exhaustion. The fourth limitation is that participants had limited to no experience in ACP with their healthcare teams (though most had experience discussing wishes with their family). This means that their perspectives on ACP may be less grounded in discernible experiences. Despite these limitations, participants were asked to reflect on what they would expect from the ACP process and on what, in an ideal situation, ACP would do for them and their loved one. In this way, our data collection resulted in a rich dataset that provide important insights into how people with frailty experience view ACP and what they hope to achieve from the process.

## 5. Conclusions

In conclusion, we sought to explore patient- and family-relevant outcomes in relation to ACP discussions. We found three broad categories of outcomes that should be further investigated and refined in future research, including research that explores the influence of people’s unique characteristics (e.g., age, comorbidities, culture) on ACP expectations. These outcomes may also be used, at least as a starting point, by those leading ACP programs and initiatives to guide planning and continuous improvement efforts. Ultimately, research and clinical programs should tailor their ACP initiatives and evaluation metrics to align with what patients and families deem most important, given they are the end recipients and intended beneficiaries of this intervention.

## Data Availability

The data presented in this study are available on request from the corresponding author due to privacy and ethical restrictions.

## Supplementary Materials

The following supporting information can be downloaded at: https://www.mdpi.com/article/10.3390/healthcare14010002/s1, File S1: Consolidated criteria for reporting qualitative studies (COREQ): 32-item checklist; File S2: Draft interview guides.
