# Supplementary material for: What Do Older Adults with Frailty and Their Caregivers Want from Advance Care Planning Discussions? A Descriptive Qualitative Study
Source: Healthcare (Basel). 2025 Dec 19;14(1):2. doi: 10.3390/healthcare14010002 (PMC12785433; doi:10.3390/healthcare14010002)
Supplement: Supplementary file 1 [file healthcare-14-00002-s001.zip › File S1 Consolidated criteria for reporting qualitative studies (COREQ) 32-item checklist.pdf]

Consolidated criteria for reporting qualitative studies (COREQ): 32-item checklist

No	Item	Guide questions/description	Author response
**Domain 1: Research team and reflexivity**			
Personal Characteristics			
1.	Interviewer/facilitator	Which author/s conducted the interview or focus group?	CK, EH, and JV; stated in text (methods section, pg 4)
2.	Credentials	What were the researcher's credentials? *E.g. PhD, MD*	RU has a PhD and an experienced qualitative researcher with >15 years of experience; CK has a PhD and an experienced qualitative researcher with >10 years of experience; JV is a Master's trained researcher, trained in qualitative methods by RU; EH is a medical student, trained in qualitative methods by RU. Training of EH and JV began with mock interviews of research staff and included regular meetings after each early interview to review transcripts and discuss the interview approach and techniques to facilitate skill development.
3.	Occupation	What was their occupation at the time of the study?	RU: Associate Professor, Department of Community Health and Epidemiology<br>CK: Project Coordinator and PhD Student, Dalhousie University<br>JV: Research Assistant and Master's student, Dalhousie University and

			Nova Scotia Health<br>EH: Research Assistant, Dalhousie University and Nova Scotia Health
4.	Gender	Was the researcher male or female?	CK and JV are female; EH is male
5.	Experience and training	What experience or training did the researcher have?	RU: PI, expertise in qualitative research<br>CK: PhD student, expertise in qualitative research<br>JV: Master's student, working for PI on various studies, including qualitative studies<br>EH: Research assistant, working for PI (and other PIs) on various studies, including qualitative studies
Relationship with participants			
6.	Relationship established	Was a relationship established prior to study commencement?	There was no relationship between the interviewers and any participants prior to study commencement
7.	Participant knowledge of the interviewer	What did the participants know about the researcher? e.*g. personal goals, reasons for doing the research*	The participants would have known nothing about the researchers prior to the study; all would have understood the reasons for doing the research as part of the informed consent process
8.	Interviewer characteristics	What characteristics were reported about the interviewer/facilitator? e.g. *Bias, assumptions, reasons and interests in the research topic*	No characteristics are reported about the interviewer. The interviewers were a PhD student (with >10 years of experience in qualitative methods), and a Master's student and Research Assistant, both supervised by the PI, an experienced qualitative researcher.
**Domain 2: study**			

**design**			
Theoretical framework			
9.	Methodological orientation and Theory	What methodological orientation was stated to underpin the study? *e.g. grounded theory, discourse analysis, ethnography, phenomenology, content analysis*	This study was a qualitative descriptive study, following the approach of Sandelowski (2000), as cited in the manuscript on page 2.
Participant selection			
10.	Sampling	How were participants selected? *e.g. purposive, convenience, consecutive, snowball*	Participants were recruited by distributing study information through patient and caregiver organizations and networks across Canada. Interested persons were instructed to contact a research coordinator for further information; stated in text (methods section, pg 2)
11.	Method of approach	How were participants approached? e.*g. face-to-face, telephone, mail, email*	Interested persons approached the research coordinator by email or telephone; this same method was then used to communicate with the interested person; stated in text (methods section, pg 2)
12.	Sample size	How many participants were in the study?	9; stated in text (results section, pg 4)
13.	Non-participation	How many people refused to participate or dropped out? Reasons?	All individuals who reached out and agreed to be interviewed took part in this study (no individuals dropped out).
Setting			
14.	Setting of data collection	Where was the data collected? e.*g. home, clinic, workplace*	In person, or via telephone or videoconferencing technology, as per the location and preference of the

			participant; stated in text (methods section, pg 3).
15.	Presence of non-participants	Was anyone else present besides the participants and researchers?	No.
16.	Description of sample	What are the important characteristics of the sample? *e.g. demographic data, date*	We have included participant type (older adult, caregiver) and gender in the results section (pg 4). Unfortunately, we did not collect participant age or SES variables for this study.
Data collection			
17.	Interview guide	Were questions, prompts, guides provided by the authors? Was it pilot tested?	Interview questions and prompts were crafted by RU and CK (both have methods expertise in semi-structured interviews). The interview guide was not pilot tested.
18.	Repeat interviews	Were repeat interviews carried out? If yes, how many?	No; stated in text (methods section, pg 3).
19.	Audio/visual recording	Did the research use audio or visual recording to collect the data?	Yes, audio recording; stated in text (methods section, pg 3).
20.	Field notes	Were field notes made during and/or after the interview or focus group?	Yes; stated in text (methods section, pg 3).
21.	Duration	What was the duration of the interviews or focus group?	Interview duration ranged from 23min:16sec to 41min:29sec.
22.	Data saturation	Was data saturation discussed?	We heard similar concepts in each interview, and across older adults with frailty and their caregivers. However, as noted in the Discussion section, we struggled to recruit older adults with frailty. Therefore, we may have gained additional perspectives and new

			concepts if we were able to interview more older adults themselves.
23.	Transcripts returned	Were transcripts returned to participants for comment and/or correction?	No.
**Domain 3: analysis and findings**			
Data analysis			
24.	Number of data coders	How many data coders coded the data?	One researcher [JV] coded all the transcripts, with another researcher [RU] coding a subset of transcripts; stated in text (methods section, pg 3).
25.	Description of the coding tree	Did authors provide a description of the coding tree?	A coding framework containing code definitions was developed by JS and RU. This was achieved through review of two interview transcripts and iterative discussion; stated in text (methods section, pg 3).
26.	Derivation of themes	Were themes identified in advance or derived from the data?	Derived from the data; analysis process discussed in text (methods section).
27.	Software	What software, if applicable, was used to manage the data?	NVIVO 12 was used to manage the coding process; stated in text (methods section, pg 3).
28.	Participant checking	Did participants provide feedback on the findings?	No.
Reporting			
29.	Quotations presented	Were participant quotations presented to illustrate the themes / findings? Was each quotation identified? e.*g. participant number*	Yes.
30.	Data and findings consistent	Was there consistency between the data presented and the findings?	Yes.

31.	Clarity of major themes	Were major themes clearly presented in the findings?	Yes.
32.	Clarity of minor themes	Is there a description of diverse cases or discussion of minor themes?	Minor themes are described in text form within the major themes.
