# Supplementary material for: What Do Older Adults with Frailty and Their Caregivers Want from Advance Care Planning Discussions? A Descriptive Qualitative Study
Source: Healthcare (Basel). 2025 Dec 19;14(1):2. doi: 10.3390/healthcare14010002 (PMC12785433; doi:10.3390/healthcare14010002)
Supplement: Supplementary file 1 [file healthcare-14-00002-s001.zip › File S2 Draft interview guides .pdf]

Draft interview guides (questions to be adapted as needed)

Questions for Older Adults Experiencing Declining Health

1. Tell me about your health situation right now.

2. What are some of the big challenges you face in terms of your health and well-being now?
   a. Physical concerns (e.g., pain, shortness of breath)
   b. Psychological / emotional concerns (e.g., anxiety, depression)
   c. Health care (e.g., accessing services, navigating the system)
   d. Social care (e.g., accessing community / volunteer services)
   e. Activities of daily living

3. What is your understanding of advance care planning?

4. Let's think about advance care planning and conversations with your health care team around goals of care as a person's health declines.

[*PROVIDE DESCRIPTION OF ADVANCE CARE PLANNING*]

a. In an ideal situation, what would advance care planning *do* for people who are experiencing declining health? What would it address? [Put another way, what would you *expect from* advance care planning?]

Alternative wording: In an ideal situation, what would advance care planning *do* for *you*? How do you think it would help with concerns identified in #2?

b. Would this change depending on where the person is in terms of declining health or illness?

Alternative wording: Would your expectations change if your health worsened?

c. In an ideal situation, what would advance care planning *do* for a person's family and friend caregivers?

Alternative wording: If you were to have an advance care plan in place, how do you think that might be helpful or beneficial to your family?

5. Is there anything else you would like to share about your thoughts on advance care planning that we haven't talked about? Or something you would like to expand on?

Questions for Family / Friend Caregiver of Older Adults Experiencing Declining Health

1. Tell me your experience as a family/friend caregiver.

2. What are your biggest concerns with respect the health and well-being of your loved one?
   a. Physical concerns (e.g., pain, shortness of breath)
   b. Psychological / emotional concerns (e.g., anxiety, depression)
   c. Health care (e.g., accessing services, navigating the system)
   d. Social care (e.g., accessing community / volunteer services)
   e. Activities of daily living

3. What is your understanding of advance care planning?

4. Let's think about advance care planning and conversations with your health care team around goals of care as a person's health declines.

[*PROVIDE DESCRIPTION OF ADVANCE CARE PLANNING*]

a) In an ideal situation, what would advance care planning *do* for people who are experiencing declining health? What would it address? [Put another way, what would you *expect from* advance care planning?]

Alternative wording: In an ideal situation, what would advance care planning *do* for your loved one? How do you think it would help with concerns identified in #2?

b) Would this change depending on where the person is in terms of declining health or illness?

Alternative wording: Would your expectations re: ACP change if your loved one's health worsened, or they were approaching the end of life?

c) In an ideal situation, what would advance care planning *do* for a person's family and friend caregivers?

Alternative wording: If your loved one were to have an advance care plan in place, how do you think that might be helpful or beneficial to you?

5. Is there anything else you would like to share about your thoughts on advance care planning that we haven't talked about? Or something you would like to expand on?
